# IL-17A promotes migration and tumor killing capability of B cells in esophageal squamous cell carcinoma

**DOI:** 10.18632/oncotarget.7869

**Published:** 2016-03-03

**Authors:** Lin Lu, Chengyin Weng, Haibo Mao, Xisheng Fang, Xia Liu, Yong Wu, Xiaofei Cao, Baoxiu Li, Xiaojun Chen, Qinquan Gan, Jianchuan Xia, Guolong Liu

**Affiliations:** ^1^ Department of Medical Oncology, Guangzhou First People's Hospital, Guangzhou Medical University, Guangzhou, 510180, China; ^2^ Department of Experimental Research and State Key Laboratory of Oncology in Southern China, Sun Yat-sen University Cancer Center, Guangzhou 510060, China

**Keywords:** IL-17A, B cells, esophageal squamous cell carcinoma, recruitment, cytotoxicity

## Abstract

We have previously reported that the accumulation of IL-17-producing cells could mediate tumor protective immunity by promoting the migration of NK cells, T cells and dendritic cells in esophageal squamous cell carcinoma (ESCC) patients. However, there were no reports concerning the effect of IL-17A on tumor infiltrating B cells. In this study, we investigated the accumulation of CD20+ B cells in the ESCC tumor nests and further addressed the effect of IL-17A on the migration and cytotoxicity of B cells. There was positive correlation between the levels of CD20+ B cells and IL-17+ cells. IL-17A could promote the ESCC tumor cells to produce more chemokines CCL2, CCL20 and CXCL13, which were associated with the migration of B cells. In addition, IL-17A enhanced the IgG-mediated antibody and complement mediated cytotoxicity of B cells against tumor cells. IL-17A-stimulated B cells gained more effective direct killing capability through enhanced expression of Granzyme B and FasL. The effect of IL-17A on the migration and cytotoxicity of B cells was IL-17A pathway dependent, which could be inhibited by IL-17A inhibitor. This study provides further understanding of the roles of IL-17A in humoral response, which may contribute to the development of novel tumor immunotherapy strategy.

## INTRODUCTION

Esophageal carcinoma ranks the 8th most common cancer and 6th leading cause of cancer-related deaths worldwide [1]. Esophageal squamous cell carcinoma (ESCC), as the major pathological type, has become a major health threat to Chinese [2]. Despite great advance in the treatment strategies, the clinical outcomes of ESCC patients remained unsatisfactory [2, 3]. According to Siegel's cancer statistics, the 5-year survival rate was 49.3% for localized disease and only 2.8% for metastatic disease [4]. Among the risk factors involving in the prognosis of ESCC patients, the host immune responses, tumor infiltrating immune cells and cytokines, are of great importance [3, 5-7]. The infiltration of IL-17+ cells plays bipolar roles in tumor immunity: tumor protective immunity or pro-tumor immunity [6, 7].

IL-17A, termed as cytotoxic T lymphocyte antigen 8 (CTLA8), was mainly produced by CD4+ T cells. And this subgroup cells were defined as T helper 17 cells (Th 17 cell) [8]. Numerous studies have investigated the roles of IL-17A in inflammation, autoimmune diseases and tumors [9-12]. The studies concerning the roles of IL-17A in tumor development are still controversial [13].

Our previous studies demonstrated that the infiltration of IL-17-producing cells in the cancer nest was correlated with better overall survival (OS) of ESCC patients [14]. IL-17A could stimulate ESCC tumor cells to produce much more chemokines to recruit more immune cells to the tumor microenvironment and enhances their antitumor functions [15]. However, there are few reports concerning the effects of IL-17A on the migration and functions of B lymphocytes. Previous studies have demonstrated that IL-17 could recruit B cells in respiratory tissues [16, 17]. However, whether IL-17 could promote the recruitment and tumor lysis of B cells in cancer remains unknown.

Thus, in this study, we investigated the accumulation and clinical significance of B cells in the ESCC tumor nests. Furthermore, we will study the effects of IL-17A on the migration and cytotoxicity of B cells. This study will provide novel experimental evidence for the underlying mechanisms of IL-17A-mediated tumor protective immunity.

## RESULTS

### Accumulation and prognostic value of CD20+ B cells in ESCC patients

**Figure 1:**
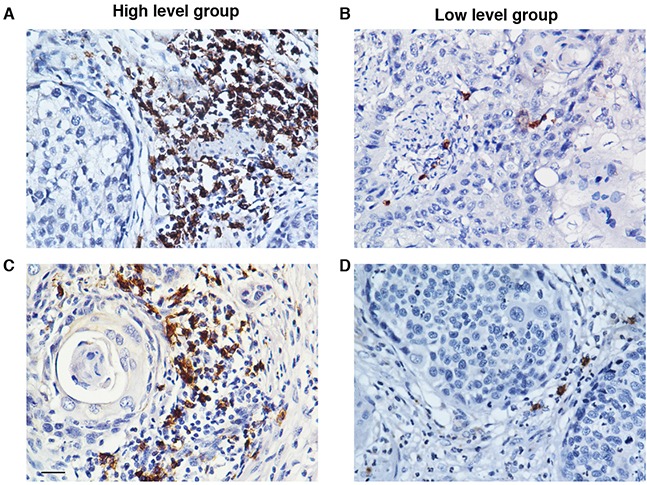
The accumulation of CD20+ B cells in the tumor nests of ESCC patients The CD20+ B cells were detected by using immunohistochemistry. Based on the median count of CD20+ B cells, the patients were divided into two groups: high CD20+ B cell group **A.** and low CD20+ B cell group **B.** (A) and (B) show the representative immunohistochemical staining photomicrographs of CD20+ B cells. Original magnification: X 400.

CD20 has been generally used as an immunohistochemical marker to detect B cell infiltration in solid tumor tissues [18-20]. Thus the accumulation of CD20+ B lymphocytes in the ESCC tumor nests was detected by IHC analysis. The representative micrographs are shown in Figure 1. The ESCC patients were divided into high or low CD20+ B cell group based on the median count of the CD20+ B cells (6.2 cells/HPF, range: 0-34.7 cells/HPF) as previously described [14].

Then we assessed the associations between the CD20+ B cell infiltration and clinico-pathological parameters of ESCC patients. As demonstrated in Table 1, the counts of CD20+ B cells were inversely associated with tumor length (*P* = 0.009) and T (tumor invasion depth) stage (*P* = 0.014).

**Table 1:** Relationship between the levels of CD20+ B cells and clinicopathologic parameters of patients with ESCC

Parameters	N of patients	CD20+ B cells		*P* value
		Low level group (N=91)	High level group (N=90)	
**Age**				0.812
**<60**	105	52	53	
**≥60**	76	39	37	
**Gender**				0.163
**Male**	141	67	74	
**Female**	40	24	16	
**Tumor length**				0.009*
**<5cm**	75	29	46	
**≥5cm**	106	62	44	
**Differentiation**				0.673
**G1**	45	24	21	
**G2**	85	44	41	
**G3**	51	23	28	
**Location**				0.253
**Upper third**	13	8	5	
**Middle third**	114	52	62	
**Lower third**	54	31	23	
**T stage**				0.014*
**T1+T2**	57	21	36	
**T3+T4**	124	70	54	
**N stage**				0.816
**No**	101	50	51	
**Yes**	80	41	39	
**M stage**				0.682
**M0**	175	87	88	
**M1**	6	4	2	
**TNM staging**				0.235
**Stage I-II**	117	55	62	
**Stage III-IV**	64	36	28	

* statistical significance, *P*<0.05; G1, well differentiated; G2, moderate differentiated; G3, poor differentiated; T, primary tumor invasion depth; N, regional lymph node; M, distant metastasis.

**Figure 2:**
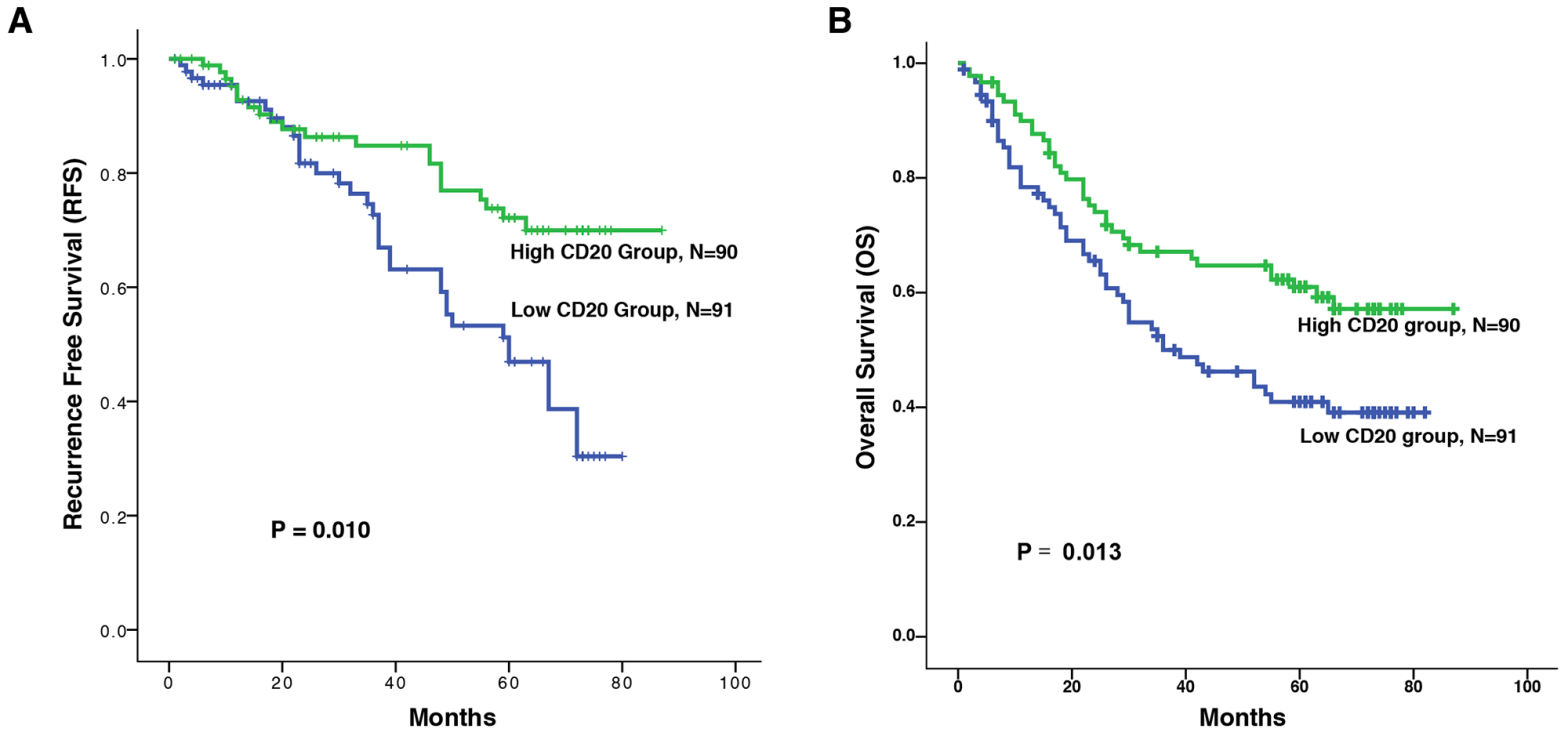
Kaplan-Meier survival analysis of CD20+ B cells in patients with ESCC Relationships between the levels of CD20+ B cells and recurrence free survival (RFS) and overall survival (OS). **A.** Increased counts of CD20+ B cells predict better RFS. **B.** Increased counts of CD20+ B cells predict better OS. The recurrence free survival (RFS) was defined as the interval between the date of surgery and date of recurrence or the last known follow-up. And the overall survival (OS) was defined as the interval between the date of surgery and date of death or the last known follow-up.

We compared the association between CD20+ B cell infiltration and the recurrence-survival rate (RFS) and overall survival (OS) of ESCC patients. There was positive correlation between the levels of CD20+ B cells and the RFS (Figure 2A, *P* = 0.010) or OS (Figure 2B, *P* = 0.013). Besides. The patients in the high CD20+ B cell group had a better RFS or OS than the patients in the low CD20+ B cell group.

The univariate analysis and subsequent multivariate analysis demonstrated that CD20+ B cells (*P* = 0.032), N stage (*P* < 0.001) and differentiation (*P* = 0.009) could be viewed as independent predictors for ESCC patients (Table 2).

**Table 2:** Univariate and multivariate analyses of variables associated with overall survival

Parameters	Univariate analysis			Multivariate analysis		
	HR	95%CI	*P* value	HR	95%CI	*P* value
**CD20+ (low/high)**	1.7061	1.113-2.616	0.014*	0.488	0.227-4.611	0.032*
**Age (≥60/<60)**	1.086	0.712-1.657	0.702			
**Gender (female/male)**	0.785	0.467-1.319	0.360			
**Location (lower/middle/upper)**	1.111	0.763-1.617	0.583			
**Length (≥5/<5)**	1.178	0.763-1.809	0.454			
**Differentiation (G3/G2/G1)**	1.554	1.150-2.100	0.004*	1.498	1.105-2.031	0.009*
**T stage (T3+4/T1+2)**	2.152	1.280-3.619	0.004*	0.481	0.272-3.111	0.078
**N stage (Yes/No)**	3.250	2.100-5.078	0.000*	1.095	0.227-7.289	< 0.001*
**M stage (M1/M0)**	2.385	0.967-5.886	0.059			
**IL-17+ (high/low)**	0.633	0.416-0.964	0.033*	0.386	0.225-2.943	0.086

### Relationships between CD20+ B cells and IL-17-producing cells in the same tumor microenvironment

**Figure 3:**
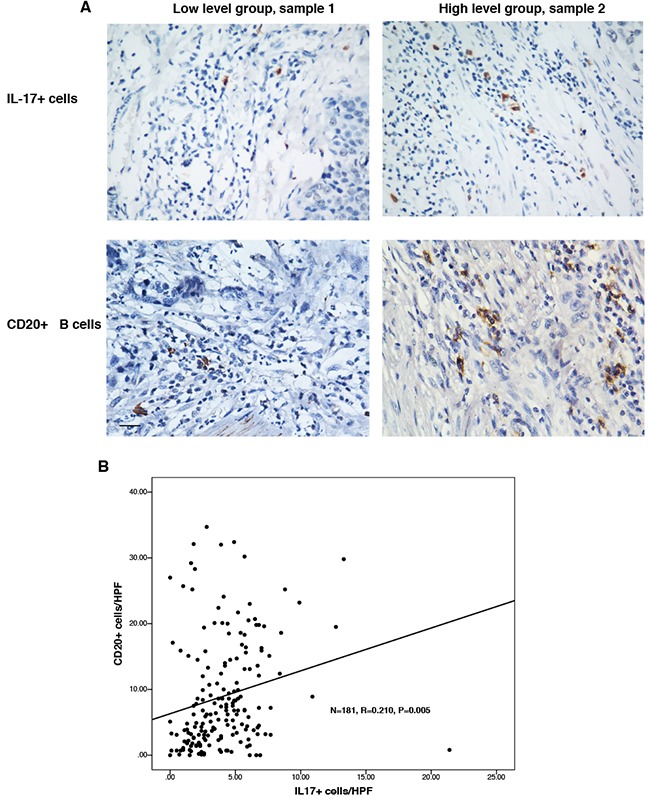
There was positive relationship between the counts of CD20+ B cells and IL-17-producing cells The numbers of CD20+ B cells and IL-17+ cells were detected using immunohistochemistry. **A.** Representative micrographs of IL-17+ TILs and CD20+ B cells in the same ESCC tissues (Left panel: sample 1; right panel: sample 2). **B.** The correlation between the counts of IL- 17A-producing cells and CD20 + B cells was determined using Pearson correlation coefficient and linear regression analyses. Original magnification: X 400.

To study the relationship between the counts of IL-17+ TILs and CD20+ B cells in the same tumor microenvironment, IHC was employed to detect the accumulations of both cells in serial tissue slides from the same tissue blocks. The representative microscopy photos of CD20+ B cells and Il-17+ cells in the same tissue block were shown in Figure 3A. We found that low CD20+ cell count was associated with low count of IL-17+ cells in the same tumor tissue (sample 1), while high CD20+ cell count was associated with larger count of IL-17+ cells in the tumor tissue (sample 2). As shown in Figure 3B, the counts of CD20+ B cells was positively correlated with the counts of IL-17-producing cells (N = 181, R = 0.210, *P* = 0.005).

### IL-17A stimulation of ESCC tumor cells resulted in promoting migration of B cells

**Figure 4:**
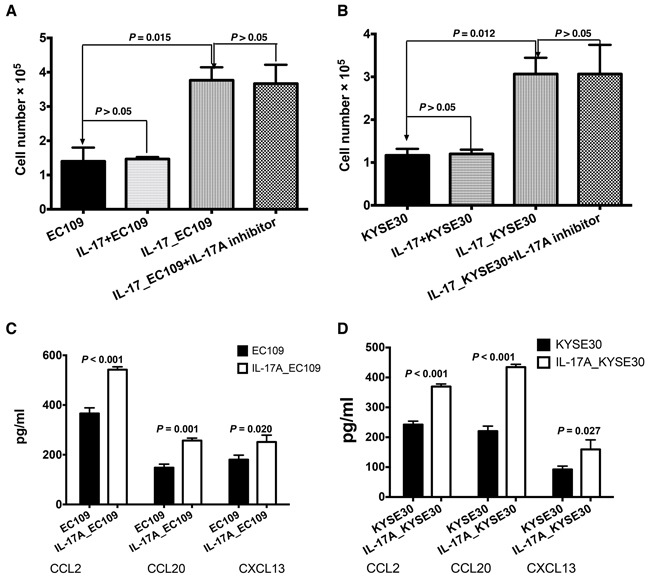
IL-17A promotes the recruitment of B cells by stimulating ESCC tumor cells to produce more chemokines **A** and **B.** The supernatants of tumor cells treated with IL-17A for 48 h (IL-17_EC109 and IL-17_KYSE30) could induce the migration of significantly higher number of B cells compared with the non-treated tumor cell supernatants (EC109 and KYSE30) or additional supplement with IL-17A (EC109+IL-17 and KYSE30+IL-17). **C.** The ELISA analysis showed that IL-17A could promote EC109 cells' production of more chemokines CCL2, CCL20 and CXCL13. **D.** Exposure to IL-17A, KYSE30 tumor cells could produce more chemokines CCL2, CCL20 and CXCL13.

To investigate whether IL-17A could recruit B cells, we performed chemotaxis assay in a chamber system. As shown in Figure 4A and 4B, supernatants from IL-17A-treated ESCC cells (IL-17_EC109 and IL-17_KYSE30) showed significantly elevated chemotaxis effects on B cells than untreated cells (*P* = 0.015 and *P* = 0.012, respectively, Figure 4A and 4B). In contrast, adding IL-17A to the supernatants from untreated ESCC cells (IL-17A+EC109 and IL-17A+KYSE30) failed to directly recruit B cells. (*P* > 0.05, Figure 4A and 4B). Moreover, additional supplement of IL-17A inhibitor secukinumab to the IL-17A_EC109 or IL-17A_KYSE 30 prevented IL-17A-mediated B cell migration (Figure 4A and 4B, *P* > 0.05), which suggesting that IL-17A pathway was required for B cell migration. These data suggested that the IL-17A might recruit B cells by stimulating tumor cells to produce some soluble factors. After stimulating with IL-17A for 24h, the levels of chemokines CCL2, CCL20 and CXCL13 were remarkably increased in both ESCC cell lines (Figure 4C and 4D, *P* < 0.05). These data suggest that IL-17A could promote the migration of B cells by stimulating the production of inflammatory chemokines from the ESCC tumor cells.

### Effect of IL-17A on the antibody and complement mediated cytotoxicity (CDC) of B cells

**Figure 5:**
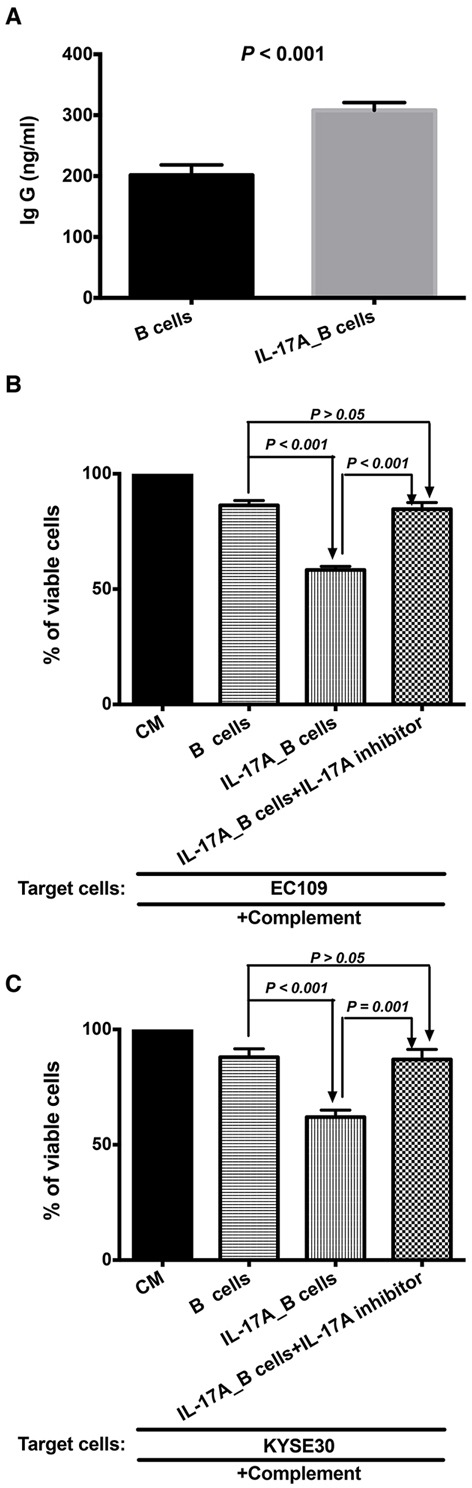
IL-17A promotes the production of IgG to enhance the antibody and complement mediated cytotoxicity (CDC) The purified B cells were treated with 50 ng/ml IL-17A for 4∼5 days (IL-17A_B cells). B cells treated without IL-17A was used as control. **A.** ELISA result shows that IL-17A stimulates the productions of IgG. IL-17A enhances the antibody and complement mediated tumor specific cytotoxicity of B cells against EC109 **B.** and KYSE 30 **C.** tumor cells. The IL-17A inhibitor could prevent the stimulation of IL-17A on the CDC of B cells.

As shown in Figure 5A, the IL-17A stimulated the B cells to produce more IgG than the control group (*P* < 0.01). The immunologic consequence of the increased production of antibody IgG was evaluated using antibody and complement mediated cytotoxicity. Immune supernatants harvested from IL-17A-treated B cells were significantly more efficient mediator of cell lysis against EC109 (Figure 5B, *P* < 0.001) and KYSE 30 (Figure 5C, *P* < 0.001). As shown in Figure 5B and 5C, additional supplement of IL-17A inhibitor could inhibit the CDC effect of IL-17A-stimulated B lymphocytes (Figure 5B, *P* < 0.001 for EC109 cells ; Figure 5C, *P* = 0.001 for KYSE30 cells).

### Effect of IL-17A on the direct cytolytic capability of B cells using LDH assay

**Figure 6:**
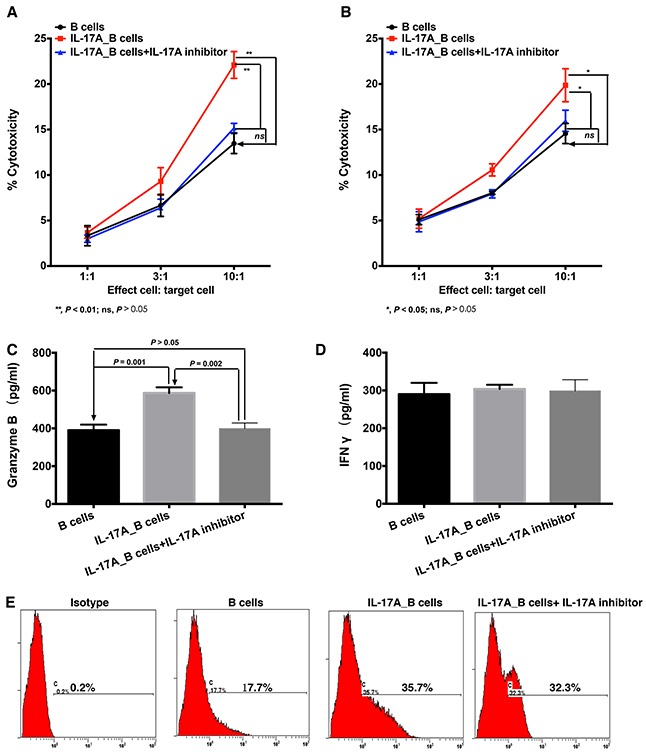
IL-17A intensifies the cytotoxicity of B cells against ESCC tumor cells through stimulating the production of cytotoxic molecules LDH assay was performed to detect the direct cytolytic capabilities of B cells against ESCC tumor cells EC109 **A.** and KYSE30 **B.** (A and B) IL-17A promotes the cytotoxicity of B cells against ESCC tumor cells. ELISA assay was performed to detect the effect of IL-17A on the production of cytotoxic molecules Granzyme B **C.** and IFNγ **D. E.** Flow cytometry analysis shows that IL-17A stimulates the expression of FasL in B cells.

Our previously study investigated that the direct cytotoxicity B cell against tumor cells could be detected by LDH assay [21]. As shown in Figure 6A and 6B, IL-17A significantly enhanced the cytotoxicity of B cells against ESCC tumor cells when the ratio of effect to target was at 10:1 (Figure 6A, *P* = 0.008 for EC109 cells; Figure 6B, *P* = 0.013 for KYSE 30 cells, respectively). Blocking IL-17A by secukinumab could inhibit the cytolytic capability of IL-17A-treated B cells (Figure 6A and 6B, *P* = 0.002 for EC109 cells and *P* = 0.033 for KYSE 30 cells, respectively).

To further investigate how IL-17A augmented the cytolytic function of B cells, we detected the expression of cytolytic molecules. We found that IL-17A significantly stimulated the production of Granzyme B in B cells (Figure 6C, *P* = 0.001), but had no effect on the production of IFN-γ (Figure 6D, *P* > 0.05). And IL-17A-stimuated Granzyme B production could be suppressed by IL-17A inhibitor secukinumab (Figure 6C, *P* = 0.002 compared to IL-17A_B cells). In addition, IL-17A treatment could also enhance FasL expression on B cells (Figure 6E). Interestingly, the IL-17A inhibitor could not significantly decrease the FasL expression (Figure 6E). These data suggested that IL-17A could stimulate B cells to produce much more cytotoxic molecules to directly target ESCC tumor cells.

## DISCUSSION

For decades, the B cells mediated-humoral immunity was overlooked. The roles played by B cells in tumor immunity are complex: either as effector cells or as antigen presenting cells [22, 23]. Activated B cells could mediated direct cytotoxicity against tumor cells. Kemp et al found that CpG-containing oligodeoxynucleotides-activated B cells could mediate lysis of tumor cells [24]. Li et al also found that LPS/anti-CD40 activated B cells could mediate direct killing of tumor cells in vitro [22]. In addition, several studies have used activated B cells as a source of effective antigen presenting cells for T cell sensitization [25, 26]. The adoptive transfer of activated B cells could exert effective anti-tumor immunity by inducing host T cell activation [22, 27]. However, adoptive transfer of B cells plus T cells could induce more efficient antitumor effect than B cells or T cells alone. And the B cell-mediated antitumor efficiency was still unsatisfactory.

In our previous studies, we found that IL-17A could mediate anti-tumor immunity by recruiting immune cells to the tumor microenvironment, such as NK cells, CD8+ T cells and CD1a+ DCs [14, 15]. As an important part of the tumor microenvironment, we found that the high B cell infiltration was significantly associated with smaller tumor diameter and lower T stage as well as better prognosis of patients, indicating a protective immunity of B cells against ESCC.

Interestingly, there was a positive correlation between the numbers of IL-17-producing cells and CD20+ B cells in the same tumor microenvironment, which indicates that the IL-17 producing cells might exert an antitumor effect by stimulating the B cells-mediated humoral response. In vitro chemotaxis assay demonstrated IL-17A could stimulate the migration of B cells. Similarly, studies in inflammatory lung tissues showed that IL-17A could promote the migration of B cells [16, 17]. Several studies have confirmed that B cells could express chemokine receptors CCR2 [28], CCR6 [29] and CXCR5 [30]. Thus, we detected the corresponding chemokines which were associated with the migration of B cells. IL-17A could stimulate ESCC tumor cells to produce much more chemokines, such as CCL2, CCL20 and CXCL13. These data suggested that one of the mechanisms underlying IL-17A-mediated antitumor immunity is mediated by recruiting an increased number of B cells to the tumor microenvironment through chemokine/chemokine receptor interactions.

One mechanism underlying B-cell mediated cytotoxicity was through antibody and complement mediated cytotoxicity. Our previous study found that cytotoxic antibody subclass IgG produced by the activated B cells could bind specifically to the tumor cells and mediate tumor cell lysis in the presence of complement [31]. We found that IL-17A promoted B cells' increased production of immunoglobulin IgG and induced much more effective antibody and complement mediated cytotoxicity (CDC). Blocking IL-17A through the anti-IL-17A antibody could inhibit the CDC effect of IL-17A_B cells, which suggested that the IL-17A pathway was involved in the CDC effect of B cells. Furthermore, LDH assay showed that IL-17A enhanced the direct cytolytic effect of B cells against ESCC tumor cells. This was due to the increased expression of Granzyme B and FasL in IL-17A-treated B cells. Our results are consistent with previous findings that there is a subpopulation of B cells which could be stimulated to produce Granzyme B [32]. Besides, Hahne et al reported that activated B cells could express FasL, which were able to kill Fas-sensitive target cells [33]. Blocking IL-17A using secukinumab could decrease the expression of Granzyme B, but FasL in IL-17A-treated B cells. These indicated that the IL-17A could enhance the indirect and direct B cell cytotoxicity by stimulating the productions of IgG and Granzyme B.

As antigen presenting cells, it is generally accepted that activated B cells could express MHC class II molecules when pulsed with exogenous antigens. And the B cell-mediated MHC class II-restricted presentation plays important roles in the establishment of humoral response [34, 35]. We hypothesized that the IL-17A_B cell-mediated cytotoxicity was MHC class II-restricted, because co-culture of ESCC tumor cell supernatant and B cells resulted in enhanced cytotoxicity. And we will further confirm this in our continuing study.

In conclusion, our study demonstrated that IL-17A could enhance the B cell-mediated humoral immunity. On one way, IL-17A could promote the migration of B cells by stimulating tumor cells to produce much more chemokines. On the other way, IL-17A could enhance the tumor killing capabilities of B cells by producing more immunogenic antibody and cytolytic molecules. Our current study would contribute to the development of more effective tumor immunotherapy.

## MATERIALS AND METHODS

### Ethical statement

The Research Ethics Committee of the Guangzhou First People's Hospital approved this study and informed consents were obtained from all participants. All experimental procedures were performed according to the Helsinki Declaration.

### Patients and specimens

Paraffin-embedded specimens were obtained from 181 cases of esophageal squamous cell carcinoma (ESCC) patients who underwent surgery between 2002 and 2003. The follow-up data of patients included in this study were more than five years, and the median follow-up time was 44 months (range: 3-87 months). All the patients included in this study were treated with surgery. And none were given with chemotherapy or radiotherapy prior to the surgery. Patients were given to chemotherapy or radiotherapy after the surgery according to the NCCN guideline. The patients were staged according to the UICC 7th stage system and graded histologically according to the WHO classification criteria.

### Cell line and culture

The ESCC cell lines EC109 and KYSE30 were purchased from the Committee of the Type Culture Collection of the Chinese Academy of Sciences (Shanghai, China), and maintained as previously described [21]. To harvest the tumor cell culture supernatant, a total amount of 2 × 10^6^ tumor cells were suspended in 4 ml CM and placed in the six-well plates. Part of the tumor cells were treated with or without 50 ng/ml IL-17A for 48 hours. Part of the tumor cells were treated with both IL-17A and IL-17A inhibitor (Secukinumab, 10 μg/ml, Novartis).

### Immunohistochemistry and scoring

The paraffin-embedded blocks were cut at a thickness of 2 μm. The slides were deparaffinized and rehydrated through graded ethanol. Then the slides were boiled in EDTA (1mM, pH 8.0) buffer in a microwave oven for the purpose of antigen retrieval as previously described [20]. After blocking the endogenous peroxidase by using 0.3% H_2_O_2_, the slides were incubated with the primary antibodies: mouse anti-human CD20 (Zhongshan Golden Bridge Company, Beijing, China; at a concentration of 1:200) or goat anti-IL-17 (R&D systems; dilution 1/100) overnight. After being incubated with HRP-conjugated secondary antibody (DAKO EnVision™ Detection Kit), the visualization signal was developed using the A solution in the DAKO kit and counterstained with hematoxylin.

The cell numbers were obtained by manually counting positively stained cells in ten randomly chosen fields under the 400× high power magnification (cells/HPF). Then the scoring was determined by computing the mean number of positively stained cells per HPF.

### B cells isolation and culture

B cells were purified from the peripheral blood of healthy donors using anti-CD19 MACS beads (MiltenyBiotec, Auburn, CA) according to the manufacture's instructions. In some experiments, the purified CD19+ B cells were activated with culture medium supplemented with IL-2 and LPS (Sigma-Aldrich, Atlanta, GA) for 4-5 days. The supernatants and B cells were collected for subsequent experiments.

### In vitro chemotaxis assay

The chemotaxis abilities of B cells were evaluated using 24-well chemotaxis chambers (5.0 μm pore size, Corning, NY) as previously described [21]. Various tumor cell culture supernatants were added in the lower chamber. About 1×10^7^ purified B cells were re-suspended in a volume of 200 μl CM and placed in the upper chamber. After 4 h incubation, the number of cells migrated into the lower chamber were counted using a hemocytometer.

### ELISA assay

The concentrations of CCL2, CCL20 and CXCL 13 (Abcam, USA) in the rumor cell culture supernatants, as well as Ig G, IFN-γ and Granzyme B in the B culture supernatants were detected using commercial ELISA (BD PharNingen, USA) kits according to the manufacturer's instructions.

### Antibody and complement mediated cytotoxicity

10^5^ viable tumor cells were incubated with immune supernatants collected from the B cells as previously described [37]. Then the cells were incubated with rabbit complement for another 1h. Finally, typan blue staining was used to analyze the cell lysis. The results was expressed as: % viable cells = the final number of viable cells/10^5^.

### LDH assay

The lactate dehydrogenase assay (LDH, CytoTox 96; Promega) was performed to detect the cytolytic abilities of B cells against ESCC tumor cells as previously described [21].

### Flow cytometry analysis

Cell surface expression of FasL was analyzed using flow cytometry as previously described [21]. Cells were stained with PE-conjugated anti-FasL purchased from BD Biosciences and analyzed on CXP Analysis software (Beckman Coulter).

### Statistical analysis

Data analysis was performed using SPSS software (version 16.0; SPSS Inc., Chicago, IL). Quantitative values were expressed as mean ± SD or median (range). The overall survival (OS) was calculated using the Kaplan-Meier method and the log-rank test. The prognostic significances were assessed by univariate and multivariate analyses using the Cox proportional hazards model. The student's *t* test was performed to compare the values between 2 groups. A two-tailed *P* value ≤ 0.05 was considered statistically significant.
